# Breakdown of holistic face processing with vertical displacement: A consequence of disrupted perceptual grouping, not biological implausibility

**DOI:** 10.3758/s13414-026-03274-z

**Published:** 2026-05-19

**Authors:** Kim M. Curby

**Affiliations:** School of Psychological Sciences, Performance and Expertise Research Centre, Macquarie University, Macquarie Park, NSW 2109 Australia

**Keywords:** Face perception, Holistic processing, Perceptual grouping

## Abstract

Holistic processing is a fast, efficient, and seemingly effortless processing style typically linked with perceiving faces and objects of expertise—where observers appear unable to selectively attend to features within a stimulus. Holistic face processing is disrupted when the top and bottom halves are vertically separated by a substantial amount. This was attributed to the biological implausibility of the elongated faces—a domain-specific explanation. However, more recent findings of face-like holistic processing of stimuli with strong perceptual grouping cues suggest that such cues support holistic processing more generally. If so, the breakdown of holistic processing with vertical separation may reflect a disruption to perceptual grouping mechanisms rather than to face-specific mechanisms. We tested this hypothesis across three experiments. Experiment 1 used a modified composite part matching paradigm to replicate previous results—vertically separating the halves in a composite face paradigm disrupted holistic face processing. Experiment 2 demonstrated that vertical part displacement similarly disrupted holistic processing of non-face stimuli rich in perceptual grouping cues, which are not constrained by biological plausibility. Experiment 3 revealed that horizontal misalignment—a standard method of disrupting holistic processing—does not further diminish holistic processing of vertically displaced non-face stimuli, consistent with the interpretation that holistic processing was already disrupted for these stimuli. These findings support a disrupted perceptual grouping, rather than biological implausibility, account of the disruption to face holistic processing with vertical part misalignment. This has implications for understanding face processing and, given the role of holistic perception in supporting perceptual expertise, skilled perception more broadly.

**Supplementary Information:**

The online version contains supplementary material available at 10.3758/s13414-026-03274-z.

Holistic processing is a characteristic processing style typically linked with perceiving faces and objects of expertise—where observers appear obliged to attend to stimuli as wholes and experience difficulty selectively attending to regions within stimuli (Farah et al., 1998; Gauthier & Tarr, 2002). Modifications to the configuration of features within stimuli impair this processing. For example, holistic face processing is disrupted when the top and bottom halves are vertically separated by a substantial amount (Taubert & Alais, 2009). This was attributed to the biological implausibility of the resulting elongated faces—a domain-specific explanation tied to the expected physical structure of faces. However, more recent findings demonstrating face-like holistic processing for novel stimuli with strong perceptual grouping cues instead support a more general account of holistic processing grounded in perceptual organisation (Curby & Moerel, 2019; Zhao et al., 2016). Specifically, the breakdown of holistic processing with vertical separation may reflect a disruption to perceptual grouping mechanisms rather than to face-specific mechanisms. The current study tests this hypothesis.

There are a number of behavioural markers of holistic processing. The composite task is one of the most used tasks to demonstrate robust behavioural characteristics of holistic face processing. This task has two versions, the original version developed by Young et al. (1987) and an extended version, referred to as the complete composite task developed later (Gauthier & Bukach, 2007). The current study uses the extended version. This task provides a behavioural index of our apparent obligation to process stimuli such as faces as wholes. In this task, participants make judgements about one half of chimeric faces composed of the top and bottom parts of different faces. Participants' performance shows evidence of interference (or facilitation) from the other, task-irrelevant half. The influence of the irrelevant face part on performance is measured by comparing performance when the relationship between the irrelevant parts is congruent versus incongruent with that between the task-relevant parts. For example, if the task-relevant parts are different, and this is also true for the task-irrelevant parts, this relationship is congruent. However, if the task-relevant parts are the same, but the task-irrelevant parts are different, this relationship is incongruent. The degree to which the congruency of the task-irrelevant part impacts judgements involving only the task-relevant part provides an index of holistic perception of the stimulus.

Highlighting the importance of the configuration of the features within faces in driving holistic processing, the influence of task-irrelevant parts in the composite task is attenuated when the configuration of the parts is disrupted, such as when the top and bottom parts are horizontally misaligned (Young et al., 1987). Similarly, other manipulations disrupting the configuration of facial features, such as rotating the face 180° (Mondloch & Maurer, 2008; Rossion & Boremanse, 2008; Yin, 1969) or scrambling the locations of the features within faces, also attenuate markers of holistic processing (Tanaka & Farah, 1993). Findings from other paradigms, such as the demonstration that facial parts are better recognised in the context of a whole face than in isolation, known as the whole–face advantage, also provides support for the importance of an intact configuration for face processing (Tanaka & Farah, 1993). Together, these findings are interpreted as demonstrating that the configuration of the features within faces is a key factor driving holistic processing.

Demonstrations of behavioural markers that appear unique to face processing, along with evidence of the importance of facial configuration in driving these markers, inspired theoretical accounts of specialised, domain-specific mechanisms. Namely, one account proposed that a rapid, obligatory detection stage relying on a coarse template of an upright face is a key initial step in face processing (Tsao & Livingstone, 2008). Specifically, it is argued that the detection of a stimulus as a face allows access to face-specific mechanisms—namely, those responsible for holistic processing, which encode faces as a unit or gestalt. Other accounts suggest that holistic processing itself is part of this early processing stage, playing a role in supporting the rapid detection of face stimuli (Taubert et al., 2011). Regardless, both accounts suggest that the detection of the first-order information characterising face stimuli—that is, the prototypical configuration of facial features, is key for holistic processing.

As noted earlier, vertically displacing the top and bottom halves of faces so they no longer abut each other also impacts holistic processing as measured in the composite face task. Specifically, Taubert and Alais (2009) demonstrated that displacing the top and bottom parts of composite faces by the equivalent of half the width of the face disrupted markers of holistic processing. However, face stimuli exhibited some degree of robustness to this manipulation; separating the parts by only a quarter-width did not disrupt holistic processing. They suggested that the configuration of parts in faces that were only displaced by quarter-width were still biologically plausible, while faces where the parts were displaced by a half-width were not. In line with domain-specific accounts of holistic face processing, the authors interpreted these findings as evidence that a requirement for holistic face processing is that faces are biologically plausible. That is, the faces must appear to be plausible examples of faces, adhering to the constraints that the human visual system expects of real faces. This account would suggest that the disruption to holistic processing when the top and bottom parts of face stimuli are vertically displaced is a result of these distorted, ecologically invalid stimuli no longer being able to activate an internal face template.

In contrast with domain-specific accounts of holistic processing suggesting that holistic processing is tied to the expected structure of faces, face-like holistic processing has been demonstrated for novel line patterns rich in perceptual grouping (gestalt) cues (Zhao et al., 2016). Notably, markers of holistic processing for these stimuli in the composite task were abolished when the gestalt cues were degraded, in this case by replacing the continuous lines within the stimuli with dotted lines. These findings challenge accounts of holistic processing that rely on domain-specific mechanisms; these stimuli engaged holistic processing despite sharing no face-like characteristics.

Given that holistic processing has been demonstrated in different contexts (e.g., amongst perceptual experts) and for different stimuli (e.g., novel line patterns) beyond faces, it is unlikely to be monolithic. However, the degree of overlap in mechanisms across different contexts and stimuli is unclear. To contribute to addressing this gap, Curby and Moerel (2019) investigated whether there is an overlap in the mechanisms supporting holistic processing for faces and stimuli rich in gestalt cues. This possibility was investigated using a modified composite part matching task where line stimuli were overlaid on face stimuli so both stimuli were present simultaneously (Curby & Moerel, 2019). Holistic processing was attenuated—that is, there was a reduced effect of congruency, when faces were overlaid with intact (aligned) line patterns, compared to misaligned line patterns, suggesting that the intact stimuli competed for the same holistic processing resources. A second experiment, examining whether holistic processing of the line patterns would be similarly impacted by overlaid face stimuli, provided support that the competition was reciprocal. Thus, faces and these stimuli with strong gestalt cues appear to share overlapping, capacity-limited holistic processing mechanisms (but see Curby et al., 2019, for evidence of a difference between this overlap and that which face processing shares with the processing of objects of expertise).

The presence of holistic processing of stimuli rich in gestalt cues provides an alternative account of the reduced holistic processing of face stimuli when the configuration of parts is disrupted. Instead of it being a result of the reduced ability of these stimuli to match an internal face template gating specialised mechanisms supporting holistic processing, it may be due to a breakdown in perceptual grouping cues within these stimuli. Consistent with this possibility, there is evidence that the disruption to holistic processing when stimulus parts are horizontally misaligned is at least in part due to the impact of this manipulation on the perceptual grouping of the parts (Curby & Entenman, 2016; Curby et al., 2013, 2016).

Evidence of the importance of more general stimulus-based factors, such as perceptual grouping, in supporting holistic processing suggests an alternative account of the disruption to holistic processing with the vertical displacement of the parts. Specifically, it is possible that rather than being a consequence of the faces no longer being biologically plausible, it arises via a disruption to the perceptual grouping of the parts. Experiment 1 aims to replicate the ability of vertical displacement to disrupt holistic face processing, while extending this finding to the complete (extended) version of the composite task paradigm. Experiment 2 will then use the same paradigm, but with holistically processed line pattern stimuli rich in gestalt cues. Notably, these novel line patterns are not constrained by notions of biological plausibility, so if the pattern of findings for faces is replicated with these stimuli, this would be inconsistent with a biological (im)plausibility account of the disruption to holistic processing with vertical part displacement. This pattern would instead be consistent with a more general disruption to gestalt grouping driving this effect. Experiment 3 will then further explore the nature of the disruption to holistic processing with vertical part displacement by investigating the relationship between the disruption caused by horizontal part misalignment (as in the classic composite task) and that caused by vertical part displacement. 

## Experiment 1

### Methods

#### Participants

Fifty-one participants were recruited with the goal of achieving a final minimum sample size of 40 after a priori exclusion criteria are applied. This sample size was chosen after a power analysis was conducted with a predicted small–medium effect size (*f* = 0.22; approximately 0.05 η_p_^2^) and a desired power of at least .85 (see preregistration: https://osf.io/2ud4x/). Recruitment took place via an online research participation pool containing Macquarie University psychology undergraduate students who received course credit for participation. All participants reported normal or corrected-to-normal vision and gave informed consent prior to participating.

#### Stimuli

The stimuli consisted of 12 greyscale front-view images of male faces wearing neutral expressions from the Karolinska Directed Emotional face (KDEF) database (Lundqvist et al., 1998). The images were cropped to remove the hair and ears and were cut in half to obtain a top and bottom half (each part was 4.4° × 3.2° of visual angle). Four additional face stimuli were used in the practice trials.

#### Design and procedure

The experiment was conducted online, and stimuli were presented via a web browser. Participants used their own devices (e.g., desktop computers, laptops, tablets). The experiment was programmed using PsychoPy and hosted by Pavlovia.

**Fig. 1:**
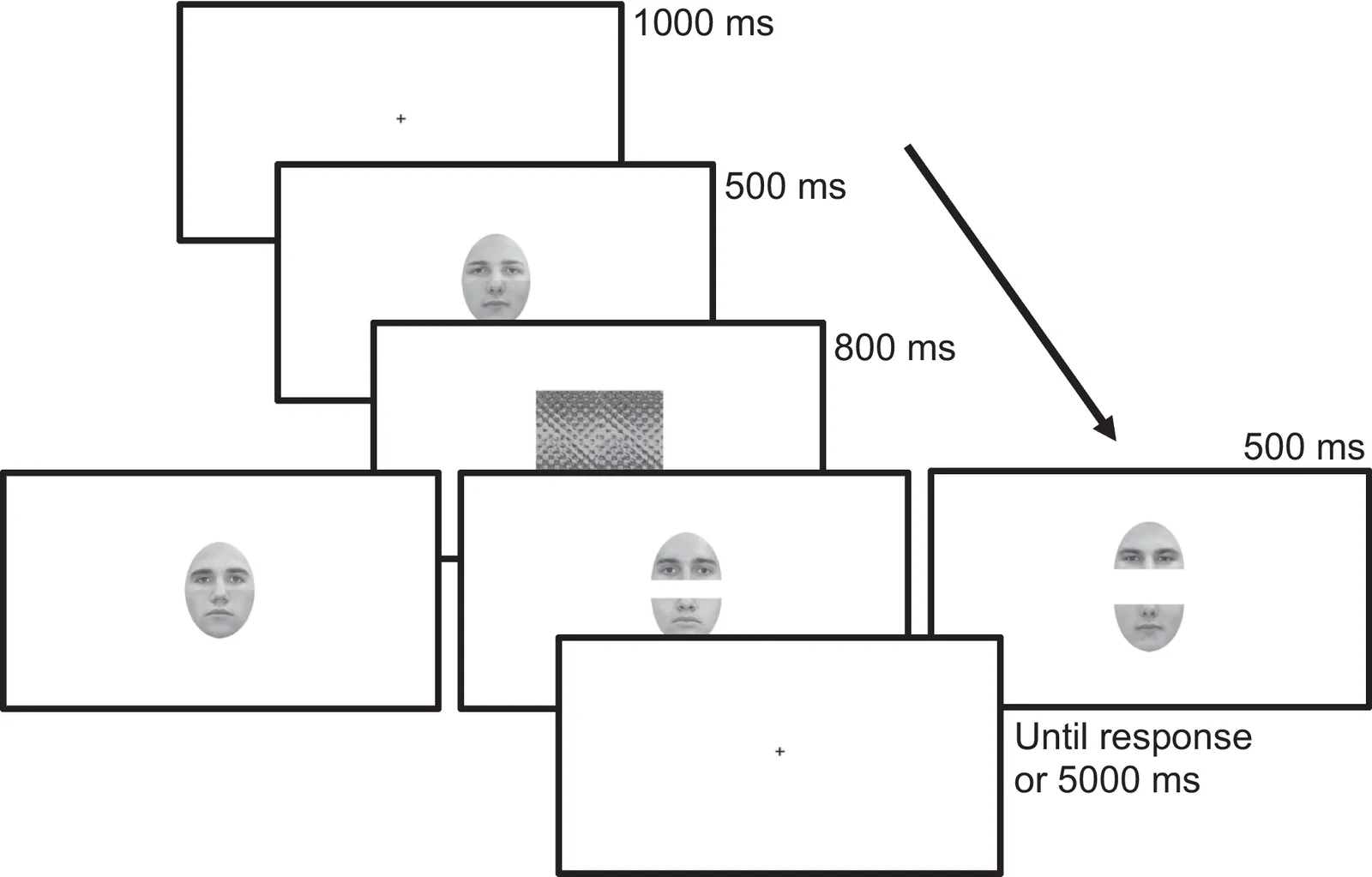
Trial structure used for the modified composite face task. Across trials, the relationship between the task-irrelevant bottom parts were either congruent or incongruent with that between the task-relevant top parts. Depending on the condition, the parts in the second composite stimulus either abutted each other or were vertically displaced by the equivalent of ¼ or ½ the width of the face

After providing informed consent, participants completed the experiment in one session of approximately 40–45 min. Each trial began with a fixation cross (1,000 ms), followed by a composite (chimeric) stimulus consisting of the top and bottom parts of different faces. After 500 ms, the stimulus was masked by a textured pattern, shown for 800 ms, before a second composite stimulus was presented (500 ms; Fig. 1). Participants were required to indicate via a keypress whether the top half of the second face was the same or different from the first. A fixation cross was presented during the response window.^1^ If no response was registered within 5 s, the next trial began automatically. The two parts of the face were either aligned, forming an intact face configuration (one-third of trials), or displaced vertically so that the face parts were either separated by the equivalent of a quarter (one-third of trials), or half (one-third of trials), of the width of the face.

In half of the trials, the same/different relationship between the task-irrelevant (bottom) stimulus parts in the two stimuli was congruent with the relationship between the task-relevant (top) parts. That is, in congruent trials, if the top parts differed between the two stimuli, thus rendering the correct response for the trial ‘different’, the bottom stimulus parts also differed. In the other trials, the same/different relationship between the task-irrelevant (bottom) stimulus parts in the two stimuli was incongruent with the relationship between the task-relevant (top) parts. For example, if the top parts of the two stimuli were the same, thus rendering the correct response for the trial ‘same’, the bottom stimulus parts differed. To ensure participants understood the task, they started with a practice block of 24 trials. The actual experiment consisted of 18 blocks, each containing 24 trials, for a total of 432 trials. Block and trial order was randomised. Sensitivity scores (*d′*) were calculated using hit rate and false alarm rates for each condition and for each participant.

### Results and discussion

**Fig. 2:**
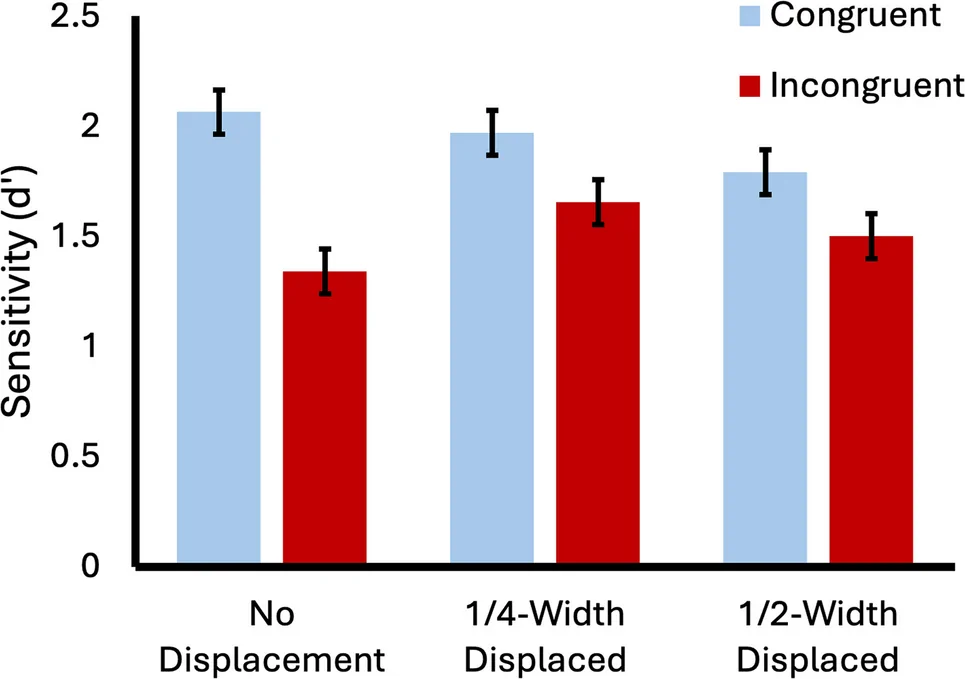
Mean sensitivity (*d′*) for the congruent (blue, pale bars) and incongruent (red, dark bars) trials in the different displacement conditions in Experiment 1. Error bars represent standard error values. (Colour figure online)

Following the exclusion criterion outlined in the preregistration, data from two participants were excluded due to failing to enter a response to at least 75% of trials, seven participants were excluded as their overall performance approximated chance-level or below (mean *d′* ≤ 0.1), and two participants were excluded due to their mean response time (RT) being greater than ±2 standard deviations from the sample mean. The remaining sample included 40 participants. The mean sensitivity scores (*d′*) and RTs (ms) for each condition for the remaining participants are presented in Fig. 2 and Table 1, respectively.

**Table 1:** Mean response times for each condition for Experiment 1

Part displacement	Intact *Mean*	*SD*	¼ width displaced *Mean*	*SD*	½ width displaced *Mean*	*SD*
*Congruent* *Incongruent*	863	21	877	22	857	23
	889	24	877	19	867	23

#### Sensitivity (*d*′)

As outlined in the preregistration, a 3 (vertical displacement; none, quarter-width, half-width) × 2 (congruency; congruent, incongruent) repeated-measures analysis of variance (ANOVA) was performed on the sensitivity data. The analysis revealed a main effect of congruency, *F*(1,39) = 16.546, *p* = .0002, η_p_^2^ = .298, with the expected higher sensitivity for congruent trials than incongruent trials, suggesting a failure to selectively attend to the task-relevant part of the faces. There was also a main effect of vertical displacement, *F*(2,78) = 3.273, *p* = .0431, η_p_^2^ = .077, and a significant interaction between vertical displacement and congruency, *F*(2,78) = 5.810, *p* = .0044, η_p_^2^ = .130 (Fig. 2). To unpack the significant interaction between displacement and congruency, follow-up standard and Bayesian paired *t*-tests comparing the size of the congruency effect across the displacement conditions were conducted (JASP, 2023).^2^ These comparisons revealed that while the size of the congruency effect did not differ between the two displaced conditions, *t*(39) = 0.174, *p* = .863, *d* = 0.027, with moderate evidence supporting this null effect (BF_01_ = 5.779), both the quarter-width displaced, *t*(39) = 2.912, *p* = .006, *d* = 0.46, and half-width displaced, *t*(39) = 3.226, *p* = .003, *d* = 0.51, conditions significantly differed from the no displacement (control) condition, with moderate (BF_10_ = 6.41) to strong (BF_10_ = 13.37) support for these differences, respectively.

#### Response time (RT)

The mean RTs for each condition (for correct trials only) are presented in Table 1. A 3 (vertical displacement; none, quarter-width, half-width) × 2 (congruency; congruent, incongruent) repeated-measures ANOVA was performed on these data to assess whether there was any evidence of a speed–accuracy trade-off that would impact the interpretation of the sensitivity data. The analysis revealed no main effect of congruency, *F*(1,39) = 1.763, *p* = .1919, η_p_^2^ = .043, or vertical displacement, *F*(2,78) < 1, or interaction between congruency and vertical displacement, *F*(2,78) < 1.

The results of Experiment 1 broadly replicate those of Taubert and Alais (2009) and extend them by using an alternative paradigm—namely, the complete version of the composite paradigm. One difference of note between the findings of Experiment 1 and those of Taubert and Alais (2009) using the partial version of the composite paradigm is that a cost to holistic processing was evident in both the quarter and half vertical displacement conditions, whereas in the previous study it was only present for the larger (half-width) displacement condition. This suggests that the complete composite paradigm may offer greater sensitivity.

## Experiment 2

Having replicated the attenuation effect of half-width vertical part displacement on holistic processing, and extending this finding to a composite paradigm including a congruency manipulation, Experiment 2 will test the hypothesis that this breakdown of holistic processing with vertical part displacement is a result of a disruption of the perceptual grouping of the face parts, rather than to a violation of biological plausibility. To do this, Experiment 2 will use the same paradigm as Experiment 1, but with line pattern stimuli that have been shown to produce the same holistic processing effects as those observed for face stimuli. If the effect of vertical displacement is a consequence of the disruption to the biological plausibility of the faces, this effect should not extend to the holistic processing of novel line patterns that are unconstrained by biological plausibility.

### Methods

#### Participants

Fifty-four participants were recruited, with the goal of having a minimum sample size of 40 after a priori exclusion criteria are applied (see Experiment 1, Methods section, for details about the power analysis and the preregistration: https://osf.io/2ud4x/). Recruitment took place via an online research participation pool containing Macquarie University psychology undergraduate students who received course credit for their participation. All participants reported normal or corrected-to-normal vision and gave informed consent prior to participating.

#### Stimuli

The stimuli set contained 12 greyscale novel line patterns created to mimic those used in Zhao et al. (2016). These stimuli were divided horizontally into a top and bottom half (each part was ~ 5.2° × 4.1° visual angle). Notably, the line patterns were created so that all lines crossed the midline at the same six locations. This constraint allowed the top and bottom of any stimulus to be paired while the gestalt cue of good continuation between the parts remained intact. Four additional stimuli were used in the practice trials.

#### Design and procedure

**Fig. 3:**
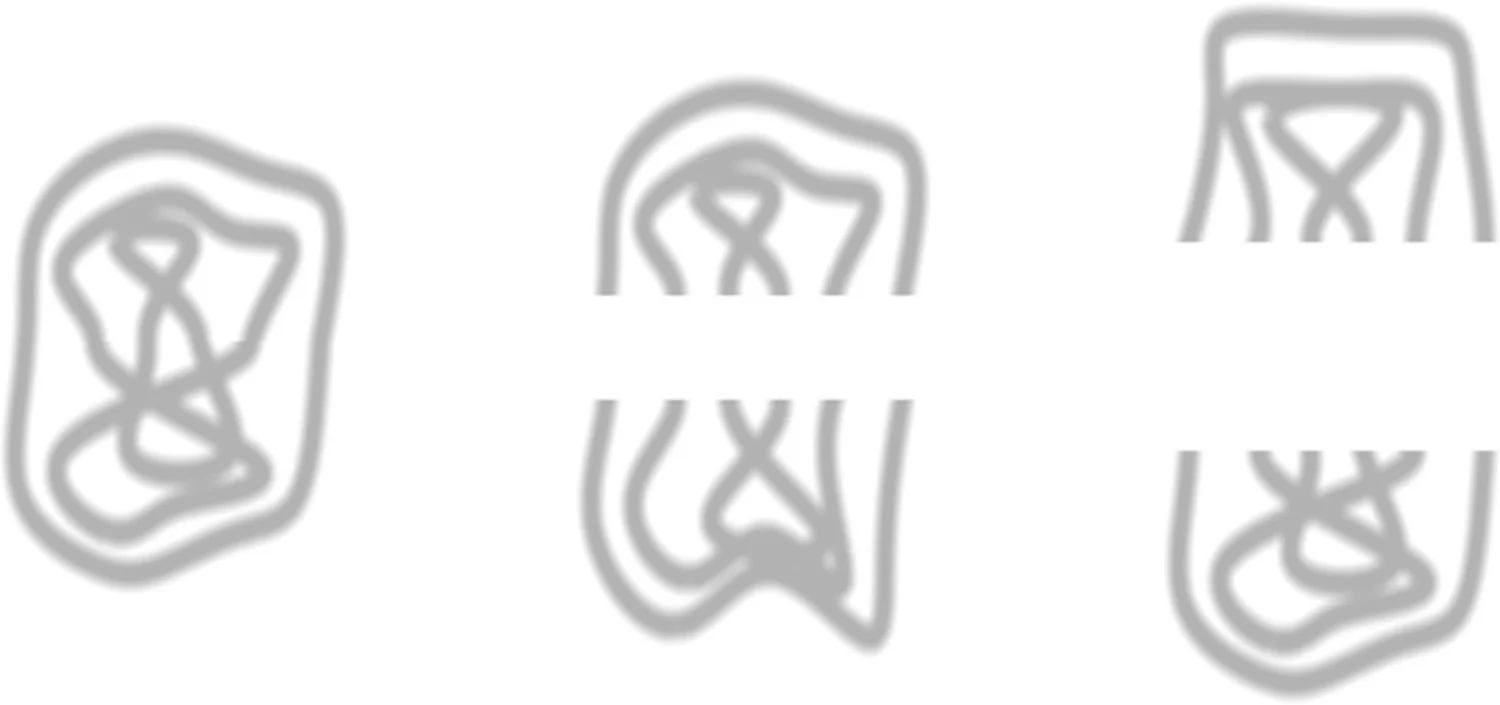
Examples of the line pattern stimuli from the three vertical displacement (none, ¼ width, and ½ width) conditions used in Experiment 2. The lines within all the stimuli intersected the horizontal midline at the same six locations, allowing any top and bottom regions of the different images to be combined without disrupting the gestalt cues within the stimuli.

The study design and procedure were the same as in Experiment 1 except that the face stimuli were replaced with line pattern stimuli (see Fig. 3 for example stimuli).

### Results and discussion

**Fig. 4:**
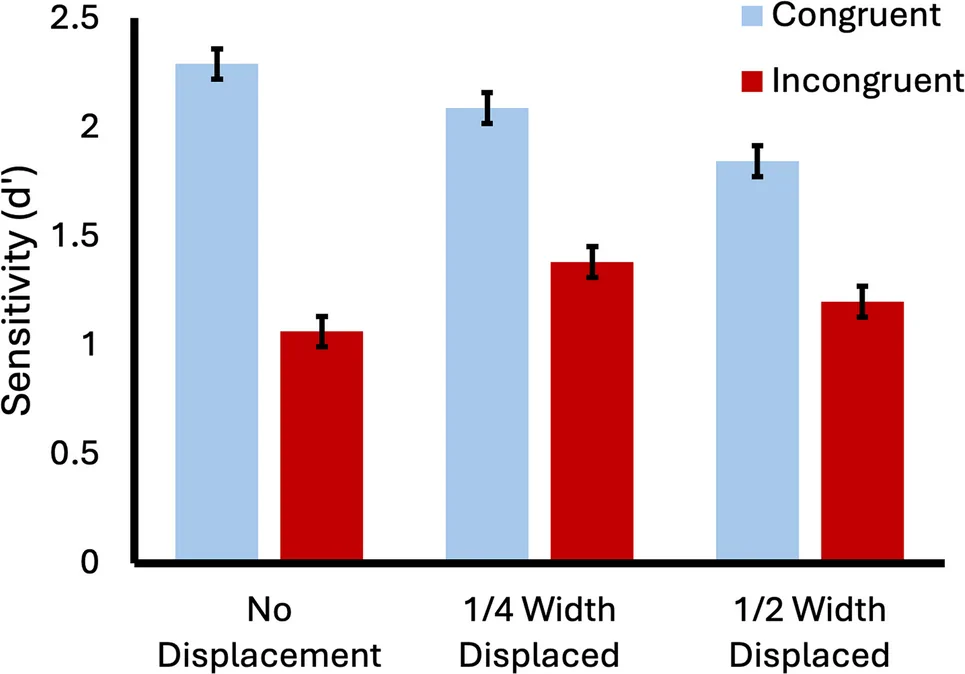
Mean sensitivity (*d′*) for the congruent (blue, pale bars) and incongruent (red, dark bars) trials in the different displacement conditions in Experiment 2. Error bars represent standard error values. (Colour figure online)

Following the preregistered exclusion criterion, data from two participants were excluded because they failed to enter a response to at least 75% of trials, seven participants were excluded because their overall performance approximated chance-level or below (mean *d′* ≤ 0.1), and no additional participants were excluded due to their mean RT being greater than ±2 standard deviations from the sample mean. The remaining sample included 45 participants. The mean sensitivity scores (*d′*) and RTs (ms) for each condition for the remaining participants are presented in Fig. 4 and Table 2, respectively.

**Table 2:** Mean response times for each condition for Experiment 2

Part displacement	Intact *Mean*	*SD*	¼ width displaced *Mean*	*SD*	½ width displaced *Mean*	*SD*
*Congruent* *Incongruent*	847	14	869	13	877	14
	888	17	899	17	929	19

#### Sensitivity (*d*′)

As outlined in the preregistration, a 3 (vertical displacement; none, quarter-width, half-width) × 2 (congruency; congruent, incongruent) repeated-measures ANOVA was performed on the sensitivity data. This analysis revealed a main effect of congruency, *F*(1,44) = 33.451, *p* ≤ .0001, η_p_^2^ = .432, with the expected higher sensitivity for congruent than incongruent trials, suggesting a failure to selectively attend to the task-relevant part of the line patterns. There was also a main effect of vertical displacement, *F*(2,88) = 4.637, *p* = .0122, η_p_^2^ = .095, and a significant interaction between vertical displacement and congruency, *F*(2,88) = 20.633, *p* ≤ .0001, η_p_^2^ = .319 (Fig. 4). To unpack the significant interaction between displacement and congruency, follow-up standard and Bayesian paired *t-*tests comparing the size of the congruency effect across the displacement conditions were conducted (JASP, 2023).^3^ These comparisons revealed that while the size of the congruency effect did not differ between the two displaced conditions, *t*(44) = 0.581, *p* = .564, *d* = 0.087, with moderate evidence supporting this null effect (BF_01_ = 5.277), both the quarter-width displaced, *t*(44) = 5.871, *p* < .001, *d* = 0.875, and half-width displaced, *t*(44) = 5.491, *p* < .001, *d* = 0.819, conditions significantly differed from the no displacement (control) condition, with very strong support for these differences (quarter-width, BF_10_ = 30,682; half-width, BF_10_ = 9,271).

#### Response time (RT)

The mean RT for correct trials for each condition is presented in Table 2. A 3 (vertical displacement; none, quarter-width, half-width) × 2 (congruency; congruent, incongruent) repeated-measures ANOVA was performed on the RT data to assess whether there was any evidence of a speed–accuracy trade-off that would impact the interpretation of the sensitivity data. The analysis revealed a main effect of congruency, *F*(1,44) = 15.531, *p* = .0003, η_p_^2^ = .261, with the expected faster response times for congruent than incongruent trials, but no main effect of vertical displacement, *F*(2,88) = 2.6905, *p* = .0734, η_p_^2^ = .058, or interaction between congruency and vertical displacement, *F*(2,88) < 1.

The results of Experiment 2 broadly replicate those of Taubert and Alais (2009) and also those of Experiment 1 with faces. Specifically, they extend this pattern of findings to holistically processed non-face novel stimuli rich in perceptual grouping cues. As in Experiment 1, a notable difference between the findings of Experiment 2 and those of Taubert and Alais (2009), using the partial version of the composite paradigm with faces, was that a cost to holistic processing was evident in both the quarter and half vertical displacement conditions. Further, the results of Experiment 1 with faces, and those of Experiment 2 with the novel line pattern stimuli, were strikingly similar, consistent with a potential common source of this effect. Notably, given the novel line patterns are unconstrained by biological plausibility, these findings are inconsistent with a biological (im)plausibility account of the disruption to holistic processing with vertical part displacement.

## Experiment 3

The findings from Experiment 2 are consistent with a disrupted perceptual grouping account of the effect of vertical displacement on holistic face processing. This account would also predict that the disruption to holistic processing should be similar in nature as that which occurs when the face parts are horizontally misaligned as both manipulations disrupt the perceptual grouping of parts. Therefore, if the effect of vertical displacement arises via a disruption to the same mechanisms that the horizontal misalignment of parts disrupts, only aligned, but not misaligned, line patterns will show an effect of vertical part displacement as these mechanisms will already be disrupted in the horizontally misaligned stimuli. To directly test this prediction, Experiment 3 will use the same paradigm as Experiment 2, but with the addition of a horizontal alignment manipulation.

### Methods

#### Participants

Fifty-six participants were recruited, with the goal of having a minimum sample size of 40 after a priori exclusion criteria are applied (see preregistration: https://osf.io/n9wj3/). Recruitment took place via an online research participation pool containing Macquarie University psychology undergraduate students who received course credit for their participation. All participants reported normal or corrected-to-normal vision and gave informed consent prior to participating.

#### Stimuli

The stimuli were the same as those used in Experiment 2.

#### Design and procedure

The design and procedure were the same as used in Experiment 2, except in half the trials, the line patterns were misaligned horizontally.

### Results and discussion

**Fig. 5:**
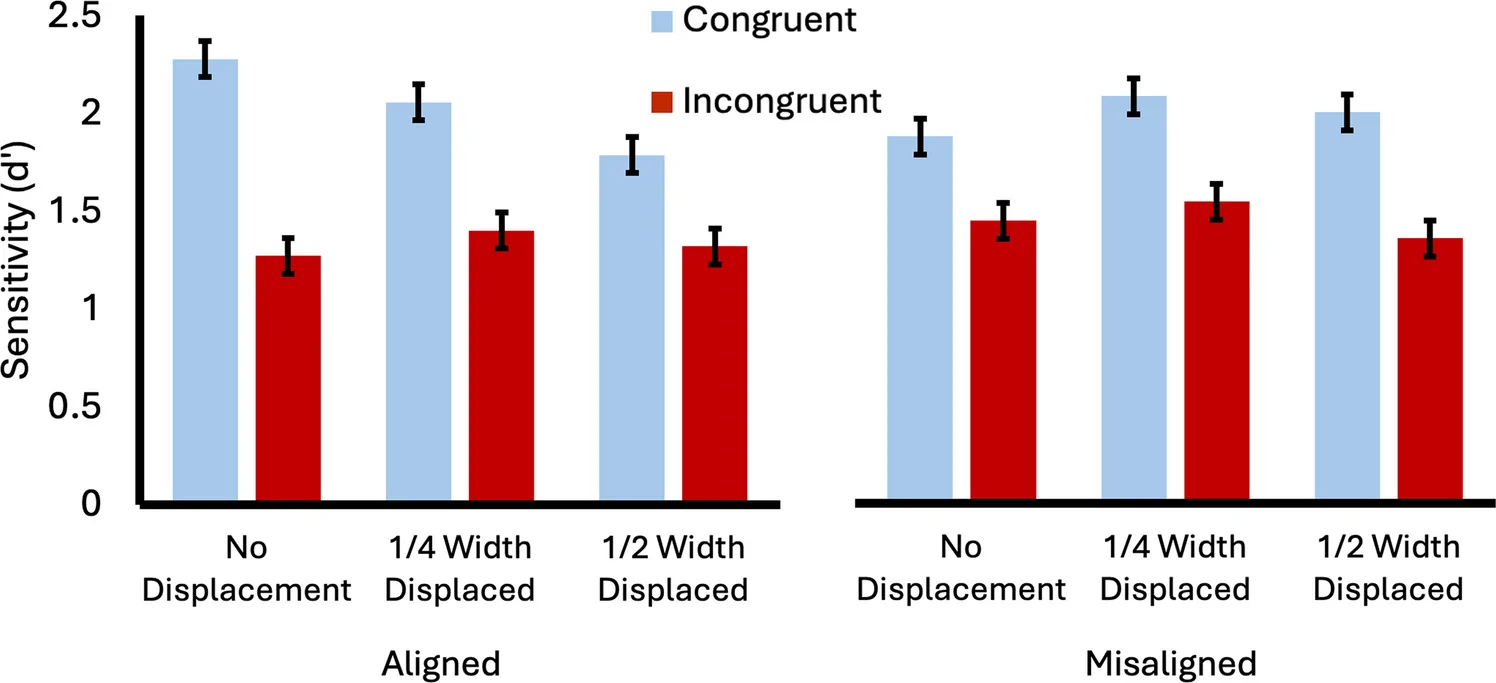
Mean sensitivity (*d′*) for the congruent (blue, pale bars) and incongruent (red, dark bars) trials in the different displacement conditions in Experiment 3 for horizontally aligned (left) and misaligned (right) stimuli. Error bars represent standard error values. (Colour figure online)

Following the exclusion criterion outlined in the preregistration, data from no participants were excluded for failing to enter a response to at least 75% of trials, 12 participants were excluded as their overall performance approximated chance-level or below (mean *d′* ≤ 0.1), and no additional participants were excluded due to their mean RT being greater than ±2 standard deviations from the sample mean. The remaining sample included 44 participants. The mean sensitivity scores (*d′*) and RT for correct trials (ms) for each condition for the remaining participants are presented in Fig. 5 and Table 3, respectively.

**Table 3:** Mean response times for each condition for Experiment 3

Part displacement	Intact *Mean*	*SD*	¼ width displaced *Mean*	*SD*	½ width displaced *Mean*	*SD*
*Aligned*						
*Congruent* *Incongruent*	839	17	882	19	876	18
	879	19	893	20	896	19
*Misaligned*						
*Congruent*	887	19	860	18	863	18
*Incongruent*	891	20	873	19	883	23

#### Sensitivity (*d*′)

As outlined in the preregistration, a 3 (vertical displacement; none, quarter-width, half-width) × 2 (congruency; congruent, incongruent) × 2 (horizontal displacement; aligned, misaligned) repeated-measures ANOVA was performed on the sensitivity data. The analysis revealed a main effect of congruency, *F*(1,43) = 25.425, *p* ≤ .0001, η_p_^2^ = .372, with the expected higher sensitivity for congruent than incongruent trials, suggesting a failure to selectively attend to the task-relevant part of the line patterns. There was also a main effect of vertical displacement, *F*(2,86) = 4.318, *p* = .0163, η_p_^2^ = .091, with Scheffé post hoc tests revealing that performance was higher in the quarter-width than the half-width condition (*p* = .019), but neither condition differed from the no displacement condition. In contrast, there was no main effect of horizontal misalignment, *F*(1,43) = 0.219, *p* = .6422, η_p_^2^ = .005. However, there was a three-way interaction between vertical displacement, horizontal misalignment, and congruency, *F*(2,86) = 8.592, *p* = .0004, η_p_^2^ = .167. To unpack this complex three-way interaction between congruency, horizontal misalignment, and vertical displacement, separate (preregistered) analyses were conducted on the trials from the horizontally aligned and misaligned conditions. This also allowed us to better probe our specific prediction—that is, that the effect of vertical displacement would only be present in the horizontally aligned conditions.

A 3 (vertical displacement; none, quarter-width, half-width) × 2 (congruency; congruent, incongruent) repeated-measures ANOVA was performed on the data from the horizontally aligned conditions revealed the expected main effect of congruency, *F*(1,43) = 21.893, *p* < .0001, η_p_^2^ = .337, with the expected higher sensitivity for congruent trials than incongruent trials. There was also a main effect of displacement, *F*(2,86) = 4.450, *p* = .0145, η_p_^2^ = .094, and a significant interaction between displacement and congruency, *F*(2,86) = 6.892, *p* = .0017, η_p_^2^ = .138 (Fig. 5). To unpack the significant interaction between displacement and congruency, standard and Bayesian paired *t*-tests comparing the size of the congruency effect across the displacement conditions were conducted (JASP, 2023).^4^ These comparisons revealed that while the size of the congruency effect did not differ between the two displacement conditions, *t*(43) = 1.404, *p* = .167, *d* = 0.212, with weak evidence supporting this null effect (BF_01_ = 2.458), both the quarter-width displaced, *t*(43) = 2.587, *p* = .013, *d* = 0.39, and half-width displaced, *t*(43) = 3.183, *p* = .003, *d* = 0.48, conditions significantly differed from the no displacement (control) condition, with moderate (quarter-width, BF_10_ = 3.119) to strong (half-width, BF_10_ = 12.267) support for these differences, respectively.

A 3 (vertical displacement; none, quarter-width, half-width) × 2 (congruency; congruent, incongruent) repeated-measures ANOVA was performed on the data from the horizontally misaligned conditions revealed a main effect of congruency, *F*(1,43) = 23.586, *p* < .0001, η_p_^2^ = .354, with higher sensitivity for congruent trials than incongruent trials. However, there was no main effect of, *F*(2,86) = 2.112, *p* = .1273, η_p_^2^ = .047^5^, or interaction with, vertical displacement *F*(2,86) = 1.222, *p* = .2998, η_p_^2^ = .028.

The absence of an interaction between congruency and vertical displacement for the misaligned line patterns in the above analysis was also examined using a Bayesian repeated-measures ANOVA conducted in JASP (2023). A model including the interaction term (congruency + displacement + congruency × displacement) was compared with a model including only the main effects. For the misaligned line patterns, the model without the interaction was 4.7 times more likely than the model including it (BF₀₁ = 4.7), providing moderate evidence against the presence of the interaction. In contrast, for the aligned line patterns, the model including the interaction was 42.3 times more likely than the model without it (BF₁₀ = 42.3), providing strong evidence for the interaction.^6^

#### Response time (RT)

A 3 (vertical displacement; none, quarter-width, half-width) × 2 (congruency; congruent, incongruent) × 2 (horizontal displacement; aligned, misaligned) repeated-measures ANOVA was performed on the RT data to assess whether there was evidence of a speed–accuracy trade-off that would impact the interpretation of the sensitivity data. The analysis revealed a main effect of congruency, *F*(1,43) = 5.218, *p* = .0273, η_p_^2^ = .108, with the expected faster response times for congruent trials than incongruent trials, consistent with a failure to selectively attend to the task-relevant part of the line patterns. However, there was no main effect of vertical displacement, *F*(2,86) = 0.171, *p* = .8429, η_p_^2^ = .004, or horizontal misalignment, *F*(1,43) = 0.022, *p* = .8810, η_p_^2^ < .001. The two-way interaction between vertical displacement and horizontal misalignment was marginally significant, *F*(2,86) = 3.293, *p* = .0419, η_p_^2^ = .037. However, Scheffé tests revealed that no comparisons between conditions were significant (all *p* values ≥ .14).

The results of Experiment 3 provide support for the effect of vertical displacement arising via a disruption to the same holistic processing mechanisms disrupted by horizontal misalignment of parts: only horizontally aligned, but not misaligned, line patterns showed an effect of vertical part displacement on the congruency effect. Specifically, this pattern suggests that these mechanisms were already disrupted by horizontally misaligning the parts.

## General discussion

Together, the findings from Experiments 1 and 2 support an account whereby the breakdown of perceptual grouping is the source of the disruption to holistic processing with the vertical displacement of face parts. Specifically, the presence of a qualitatively similar pattern of disruption to holistic processing observed for holistically processed novel line patterns in Experiment 2 as that shown for faces in Experiment 1, is inconsistent with a biological (im)plausibility account of this effect. Notably, the novel line patterns used in Experiment 2 are unconstrained by notions of biological plausibility and are instead defined by their rich gestalt cues. Given that previous work has established that these stimuli show face-like markers of holistic processing, and that these markers depend on the integrity of the gestalt cues, these findings are consistent with a disrupted perceptual grouping account of the degraded holistic processing with vertical part displacement.

The findings of Experiment 3 provide evidence that the effect of vertical displacement impacts the same mechanisms as those impaired by the horizontal misalignment of parts. Specifically, only aligned line patterns, but not misaligned ones, whose holistic processing would have already been disrupted, show an effect of the vertical displacement of their parts. Broadly, these results are consistent with the importance of fundamental perceptual grouping mechanisms in supporting holistic processing of face and non-face stimuli.

Notably, while the results from Experiment 1 broadly replicate those from Taubert and Alais’s (2009) study with faces, they were not entirely the same. Namely, while Taubert and Alais found that only the half-width vertical displacement impacted holistic processing, we found evidence of a disruption in both the quarter-width and half-width displacement conditions. Notably, while both experiments used a composite paradigm, there were considerable differences between the paradigms used that may explain the different results. Specifically, while the earlier study used the traditional composite paradigm, here we used the extended paradigm, typically referred to as the complete paradigm. Notably, a key difference between the original paradigm and this extended paradigm is that the original paradigm focuses on incongruent trials. Specifically, only trials where the task-irrelevant part is incongruent are analysed. Consistent with the possibility that the extended composite task provides greater sensitivity, this paradigm was also able to detect a disruption in both displacement conditions for line patterns in Experiment 2 and in Experiment 3. Thus, it is possible that the inclusion of both congruent and incongruent conditions in the analysis of the data from the extended paradigm increases its sensitivity and thereby increasing its ability to detect a disruption to holistic processing in the quarter-width displacement condition.

Further, Richler and Gauthier (2014) argue that the original (partial) version of the composite task is more vulnerable to response biases. They argue this makes it hard to distinguish true holistic processing from strategic or decisional biases. However, it has also been argued that the complete design may also be measuring something different—specifically, that holistic processing as a congruency effect (i.e., the performance difference between congruent and incongruent trials) risks conflating face-specific integration with general conflict/interference effects that occur in many tasks (Rossion, 2013). By contrast, they argue that the alignment effect in the partial design is more face-specific and less contaminated by general conflict-resolution processes.

Inconsistent with suggestions that the complete composite paradigm is measuring something different, the pattern of performance was qualitatively the same when condition means were recalculated only using the trials that overlap with the partial paradigm^7^. However, consistent with the increased sensitivity afforded by the complete paradigm, while the pattern was qualitatively the same, the key effects did not reach significance. Although, this is more likely due to a far smaller number of trials included in this analysis. Notably, the pattern in these means also better approximated that from Taubert and Alais’s (2009) study, where the half-width displacement appeared to have a larger impact on holistic processing than the quarter-width condition.

Another potential advantage of using the extended or complete composite task paradigm is that it can offer further insight into the contribution of facilitation versus interference to holistic processing effects. The failure to attend, and process, only the task-relevant part can differentially impact performance depending on if the other, task-irrelevant, part provides information that would sway participants’ decisions in the correct (congruent task-irrelevant part) or incorrect (incongruent task-irrelevant part) direction. Consideration of both these components can aid in our understanding of the nature of holistic processing.

Intriguingly, findings from a recent study suggest that the contribution of facilitation and interference to the congruency effect are independent (Jin et al., 2024). Specifically, Jin and colleagues (2024) found that facilitation and interference components of the congruency effect in the complete composite paradigm are differentially influenced by the location and cuing probabilities of the target half. They interpret this as evidence that these effects operate independently. Consistent with the potential independence of interference and facilitation effects, performance in the congruent conditions (facilitation) in the studies reported here show a linear-like effect of displacement, with the greater displacement producing a larger drop in this effect, while performance in the incongruent trials (interference) show a more binary-like effect, with the quarter and half displacement having a similar effect. Future studies are needed to better investigate the potential independence of the effects of vertical displacement on facilitation and interference components of holistic processing as measured in the complete composite task.

While the results reported here are inconsistent with biological plausibility being a critical precursor to holistic processing, they are consistent with a more general role of the plausibility of the stimulus as a perceptual unit. That is, the plausibility that the components belong to the same perceptual unit—namely, whether they can be successfully grouped together by foundational grouping mechanisms that govern domain-general perceptual organisation. It is this that appears critical for holistic processing to occur.

Previous findings suggest that experience can shape the perceptual plausibility of a disjointed stimulus, thereby impacting holistic processing. When the top and bottom part of faces were presented on different coloured and misaligned rectangle backgrounds, these face parts, although not misaligned themselves, were processed less holistically than those presented on aligned and uniformly coloured backgrounds (Curby et al., 2013, 2016). However, when participants completed a pre-task that established the plausibility of the two differently coloured and misaligned rectangle shapes as part of the same perceptual unit, holistic processing of face parts presented on these backgrounds was restored (Curby et al., 2016). These findings raise the possibility that it might be possible to restore holistic processing of the vertically displaced stimuli via a pretask training designed to facilitate the perception of the vertically displaced parts as being part of a single perceptual unit.

It is worth considering potential differences in the impact of the classic horizontal misalignment manipulation used to disrupt holistic processing, and the vertical displacement manipulation used in the current study, on the different configural information in faces. Two subtypes of configural information within faces have been identified, these are referred to as first-order and second-order configural information (Maurer et al., 2002). The first-order configuration of the face refers to the basic configuration that defines the stimulus as a face—that is, the presence of two eyes above a centrally placed nose, above a centrally placed mouth. In contrast, the second-order configuration is the more subtle differences in configuration that allow us to distinguish between face stimuli. While horizontal misalignment of parts impacts both first-order and second-order configural information in faces, vertical displacement of the face parts, while pushing the limits of the first-order configuration of the face, leaves it somewhat intact. That is, two eyes are still present over a centrally placed nose, above a centrally placed mouth. Notably, it is the distance between these features—that is, the second-order configural information, which is grossly distorted. Together with Taubert and Alais’s (2009) previous findings, our findings suggest that holistic processing is impaired by disrupting either the first-order and/or the second-order configural information in faces, although a larger distortion of the second-order information appears necessary to disrupt holistic processing.

At a theoretical level, domain-specific accounts have proposed that first-order and second-order configural information may serve different roles and are extracted at different temporal stages. One account suggests that face processing includes an early rapid detection phase, relying on first-order configural information that alerts the visual system to the presence of a face, stage 1, that then opens the gateway, or triggers, domain-specific holistic processing mechanisms that occur in stage 2, providing access to the second-order configural information (Tsao & Livingstone, 2008). Although, others argue that holistic processing mechanisms also operate earlier, supporting the detection of faces. For example, Taubert and colleagues (2011) suggest that holistic processing is involved in both the extraction of first-order configural information when a face is detected as well as aiding the later extraction of second-order configuration information to aid identity recognition. This suggestion is consistent with the finding that the holistic effects measured by the composite task are impacted by disrupting the first-order and second-order configural information in faces.

Alternatively, perceptual expertise accounts of holistic processing propose that it is driven by learned patterns of attention developed and automatised by extensive experience with a particular domain of stimuli (Chua et al., 2014, 2015). This pattern of learned attention typically spans multiple features and locations of the stimulus, resulting in a failure of selective attention when observers are asked to make a judgement about only a part, as in the composite task. The findings of the studies reported here suggest that vertical displacement, like the horizontal misalignment of parts, impairs the activation of these learned patterns of attention.

In the context of the dual pathway account of holistic processing effects (Curby et al., 2019; Curby & Moerel, 2019; Zhao et al., 2015, 2016), the current findings suggest that vertical displacement of parts disrupts the stimulus-based pathway to holistic processing. It is also possible that it would disrupt the experience-driven holistic processing pathway, as this manipulation would likely disrupt learned patterns of attention, given that similar disruptions to the prototypicality of objects of expertise (e.g., misalignment of parts or the inversion of the task-irrelevant part) also disrupts holistic processing of objects of expertise. However, assessing the effect of vertical displacement on the processing of objects of expertise would be necessary to assess this possibility.

A common stimulus manipulation to dissociate stimulus-based versus experience-based effects is inversion—that is, 180° rotation. An interesting question is how the processing of inverted faces might be impacted by vertical part displacement. When considering this question, it is necessary to first note that while there is evidence that inverted face processing shows some markers of holistic processing, these markers are typically delayed and reduced relative to those for upright faces (Curby & Teichmann, 2022; Richler et al., 2011). While inverted faces are fairly equivalent at a stimulus-level, they differ substantially from upright faces in terms of observers' experience; upright faces dominate observer’s experience. Thus, larger or more robust holistic processing markers for upright compared with inverted faces are typically attributed to experience. Notably, while stimulus-based perceptual grouping cues within inverted faces should still be intact, such stimuli would not benefit from the documented effects of experience in enhancing perceptual organisation and the perception of objecthood (Kimchi & Hadad, 2002; Noudoost et al., 2005; Zemel et al., 2002). Thus, inverted faces would be expected to show some disruption to holistic processing due to vertical part displacement, but it would be smaller than that for upright faces, given holistic processing is already constrained for these stimuli. However, future research is required to examine this possibility.

In conclusion, the current studies replicate and extend previous findings of the impact of vertical part displacement on markers of holistic processing in the composite face task. Specifically, we extend earlier findings by replicating them using the extended, complete version of the composite task (Experiment 1) and also by showing that the same effects can be found for non-face, novel stimuli previously shown to demonstrate face-like markers of holistic processing (Experiment 2). Further, we provide evidence consistent with vertical displacement and horizontal misalignment impacting the same holistic processing mechanisms (Experiment 3). These findings suggest that, rather than biological plausibility, it may be the plausibility of the perceptual unit more broadly that is a key precursor to holistic processing. Thus, it is the degradation of perceptual grouping more generally that disrupts holistic processing when parts are vertically displaced, whether those parts are from face or non-face stimuli. Taken together with other research emphasising the importance of fundamental stimulus properties like perceptual organisation for holistic processing, these findings suggest ways to bolster and protect this processing. In addition, at a more practical level, it also suggests ways to disrupt holistic processing if the failure of selective attention it induces is not optimal for the task at hand. Further, given the established role of holistic perception in supporting perceptual expertise, these findings have implications for understanding not only face processing, but skilled perception more broadly.

## Supplementary Information

Below is the link to the electronic supplementary material.

Supplementary file1 (DOCX 16 KB)

## Data Availability

All data and study materials are available upon request.

## Footnotes

1 Eye movements were not recorded. However, previous work has demonstrated that the markers of holistic processing revealed by the composite task emerge prior to participants having sufficient time to make an eye movement (e.g., Curby & Teichmann, 2022; Richler et al., 2011).

2 These analyses were not included in the preregistration but were conducted to aid interpretation of the null effects found in the preregistered analyses.

3 These analyses were not included in the preregistration but were conducted to aid interpretation of the null effects found in the preregistered analyses.

4 These analyses were not included in the preregistration but were conducted to aid interpretation of the null effects found in the preregistered analyses.

5 BF_01_ = 3.054 suggesting there is weak to moderate evidence for the absence of a main effect of vertical displacement.

6 These Bayesian analyses were not included in the preregistration but were conducted to aid interpretation of the null effects found in the preregistered analyses.

7 See supplementary materials for a table of these means.

## Code availability

The code to run the study is available upon request.

## Declarations

### Open practices statement

The data and materials for both experiments are available upon request. All three experiments were preregistered using an open-ended registration form via the Open Science Framework (Experiment 1& 2; https://osf.io/2ud4x/, Experiment 3; https://osf.io/n9wj3/).

## References

[CR1] Chua, K. W., Richler, J. J., & Gauthier, I. (2014). Becoming a Lunari or Taiyo expert: Learned attention to parts drives holistic processing of faces. *Journal of Experimental Psychology: Human Perception and Performance,**40*(3), 1174–1182. 10.1037/a0035895 24588261 10.1037/a0035895 PMC4153696

[CR2] Chua, K. W., Richler, J. J., & Gauthier, I. (2015). Holistic processing from learned attention to parts. *Journal of Experimental Psychology: General,**144*(4), 723–729. 10.1037/xge0000063 25775049 10.1037/xge0000063 PMC4922746

[CR3] Curby, K. M., & Entenman, R. (2016). Framing faces: Frame alignment impacts holistic face perception. *Attention, Perception, & Psychophysics,**78*(8), 2569–2578. 10.3758/s13414-016-1194-4 10.3758/s13414-016-1194-4 27590479

[CR4] Curby, K. M., Entenman, R. J., & Fleming, J. T. (2016). Holistic face perception is modulated by experience-dependent perceptual grouping. *Attention, Perception, & Psychophysics,**78*(5), 1392–1404. 10.3758/s13414-016-1077-8 10.3758/s13414-016-1077-8 27029482

[CR5] Curby, K. M., Goldstein, R. R., & Blacker, K. (2013). Disrupting perceptual grouping of face parts impairs holistic face processing. *Attention, Perception, & Psychophysics,**75*(1), 83–91. 10.3758/s13414-012-0386-9 10.3758/s13414-012-0386-9 PMC3824569 23179914

[CR6] Curby, K. M., Huang, M., & Moerel, D. (2019). Multiple paths to holistic processing: Holistic processing of gestalt stimuli do not overlap with holistic face processing in the same manner as do objects of expertise. *Attention, Perception, & Psychophysics,**81*(3), 716–726. 10.3758/s13414-018-01643-x 10.3758/s13414-018-01643-x 30569435

[CR7] Curby, K. M., & Moerel, D. (2019). Behind the face of holistic perception: Holistic processing of gestalt stimuli and faces recruit overlapping perceptual mechanisms. *Attention, Perception, & Psychophysics,**81*(8), 2873–2880. 10.3758/s13414-019-01749-w 10.3758/s13414-019-01749-w 31165455

[CR8] Curby, K. M., & Teichmann, L. (2022). The time course of holistic processing is similar for face and non-face gestalt stimuli. *Attention, Perception, & Psychophysics,**84*(4), 1234–1247. 10.3758/s13414-021-02415-w 10.3758/s13414-021-02415-w PMC9076732 35460025

[CR9] Farah, M. J., Wilson, K. D., Drain, M., & Tanaka, J. W. (1998). What is “special” about face perception? *Psychological Review,**105*(3), 482–498. 9697428 10.1037/0033-295x.105.3.482

[CR10] Gauthier, I., & Bukach, C. (2007). Should we reject the expertise hypothesis? *Cognition,**103*(2), 322–330. 10.1016/j.cognition.2006.05.003 16780825 10.1016/j.cognition.2006.05.003

[CR11] Gauthier, I., & Tarr, M. J. (2002). Unraveling mechanisms for expert object recognition: Bridging brain activity and behavior. *Journal of Experimental Psychology: Human Perception and Performance,**28*(2), 431–446. 11999864 10.1037//0096-1523.28.2.431

[CR12] JASP. (2023). *JASP* (Version 0.17.1) [Computer software].

[CR13] Jin, H., Ji, L., Cheung, O. S., & Hayward, W. G. (2024). Facilitation and interference are asymmetric in holistic face processing. *Psychonomic Bulletin & Review,**31*(5), 2214–2225. 10.3758/s13423-024-02481-9 38438710 10.3758/s13423-024-02481-9 PMC11543743

[CR14] Kimchi, R., & Hadad, B. S. (2002). Influence of past experience on perceptual grouping. *Psychological Science,**13*(1), 41–47. 11892777 10.1111/1467-9280.00407

[CR15] Lundqvist, D., Flykt, A., & Ohman, A. (1998). *The Karolinska Directed Emotional Faces-KDEF* [CD-ROM]. Department of Clinical Neuroscience, Psychology section, Karolinska Institutet, Stockholm, Sweden.

[CR16] Maurer, D., Le Grand, R. L., & Mondloch, C. J. (2002). The many faces of configural processing. *Trends in Cognitive Sciences,**6*(6), 255–260. 10.1016/s1364-6613(02)01903-4 12039607 10.1016/s1364-6613(02)01903-4

[CR17] Mondloch, C. J., & Maurer, D. (2008). The effect of face orientation on holistic processing. *Perception,**37*(8), 1175–1186. 10.1068/p6048 18853554 10.1068/p6048

[CR18] Noudoost, B., Adibi, M., Moeeny, A., & Esteky, H. (2005). Configural and analytical processing of familiar and unfamiliar objects. *Cognitive Brain Research,**24*(3), 436–441. 10.1016/j.cogbrainres.2005.02.012 16099356 10.1016/j.cogbrainres.2005.02.012

[CR19] Richler, J. J., & Gauthier, I. (2014). A meta-analysis and review of holistic face processing. *Psychological Bulletin,**140*(5), 1281–1302. 10.1037/a0037004 24956123 10.1037/a0037004 PMC4152424

[CR20] Richler, J. J., Mack, M. L., Palmeri, T. J., & Gauthier, I. (2011). Inverted faces are (eventually) processed holistically. *Vision Research,**51*(3), 333–342. 10.1016/j.visres.2010.11.014 21130798 10.1016/j.visres.2010.11.014

[CR21] Rossion, B. (2013). The composite face illusion: A whole window into our understanding of holistic face perception. *Visual Cognition,**21*(2), 139–253. 10.1080/13506285.2013.772929

[CR22] Rossion, B., & Boremanse, A. (2008). Nonlinear relationship between holistic processing of individual faces and picture-plane rotation: Evidence from the face composite illusion. *Journal of Vision,**8*(4), Article 3. 10.1167/8.4.3 10.1167/8.4.3 18484842

[CR23] Tanaka, J. W., & Farah, M. J. (1993). Parts and wholes in face recognition. *The Quarterly Journal of Experimental Psychology Section A,**46*(2), 225–245. 10.1080/14640749308401045 10.1080/14640749308401045 8316637

[CR24] Taubert, J., & Alais, D. (2009). The composite illusion requires composite face stimuli to be biologically plausible. *Vision Research,**49*(14), 1877–1885. 10.1016/j.visres.2009.04.025 19426751 10.1016/j.visres.2009.04.025

[CR25] Taubert, J., Apthorp, D., Aagten-Murphy, D., & Alais, D. (2011). The role of holistic processing in face perception: Evidence from the face inversion effect. *Vision Research,**51*(11), 1273–1278. 10.1016/j.visres.2011.04.002 21496463 10.1016/j.visres.2011.04.002

[CR26] Tsao, D. Y., & Livingstone, M. S. (2008). Mechanisms of face perception. *Annual Review of Neuroscience,**31*, 411–437. 10.1146/annurev.neuro.30.051606.094238 18558862 10.1146/annurev.neuro.30.051606.094238 PMC2629401

[CR27] Yin, R. K. (1969). Looking at upside-down faces. *Journal of Experimental Psychology,**81*(1), 141–145.

[CR28] Young, A. W., Hellawell, D., & Hay, D. (1987). Configural information in face perception. *Perception,**10*, 747–759. 10.1068/p160747 3454432

[CR29] Zemel, R. S., Mozer, M. C., Behrmann, M., & Bavelier, D. (2002). Experience-dependent perceptual grouping and object-based attention. *Journal of Experimental Psychology: Human Perception and Performance,**28*(1), 202–217.

[CR30] Zhao, M., Bulthoff, H. H., & Bulthoff, I. (2015). A shape-based account for holistic face processing. *Journal of Experimental Psychology: Learning, Memory and Cognition*. 10.1037/xlm0000185 10.1037/xlm0000185 26371495

[CR31] Zhao, M., Bulthoff, H. H., & Bulthoff, I. (2016). Beyond faces and expertise: Facelike holistic processing of nonface objects in the absence of expertise. *Psychological Science,**27*(2), 213–222. 10.1177/0956797615617779 26674129 10.1177/0956797615617779 PMC4750070

