# Supplementary material for: Breakdown of holistic face processing with vertical displacement: A consequence of disrupted perceptual grouping, not biological implausibility
Source: Atten Percept Psychophys. 2026 May 19;88(5):133. doi: 10.3758/s13414-026-03274-z (PMC13186796; doi:10.3758/s13414-026-03274-z)
Supplement: Supplementary file 1 — Supplementary file1 (DOCX 16 KB) [file 13414_2026_3274_MOESM1_ESM.docx]

Supplementary Materials

Supplementary Table 1. Mean accuracy (% correct) for each condition in Experiment 3 calculated only using trials from the partial composite design.

| **Part Displacement** | **Intact**  *Mean* | *S.D.* | **¼ Width Displaced**  *Mean* | *S.D.* | **½ Width Displaced**  *Mean* | *S.D.* |
| --- | --- | --- | --- | --- | --- | --- |
| *Aligned*  *Misaligned* | 67.9 | 25 | 70.3 | 23 | 69.1 | 23 |
|  | 72.1 | 21 | 73.0 | 11 | 69.1 | 24 |
